# Facilitators and Barriers of COVID-19 Vaccine Promotion on Social Media in the United States: A Systematic Review

**DOI:** 10.3390/healthcare10020321

**Published:** 2022-02-08

**Authors:** Cristian Lieneck, Katharine Heinemann, Janki Patel, Hung Huynh, Abigail Leafblad, Emmanuel Moreno, Claire Wingfield

**Affiliations:** School of Health Administration, Texas State University, San Marcos, TX 78666, USA; kah357@txstate.edu (K.H.); jdp285@txstate.edu (J.P.); hphuynh5@txstate.edu (H.H.); agl54@txstate.edu (A.L.); e_m814@txstate.edu (E.M.); cew161@txstate.edu (C.W.)

**Keywords:** social media, COVID-19, Coronavirus, vaccination

## Abstract

*Background and Objectives:* Information regarding the COVID-19 pandemic has spread internationally through a variety of platforms, including social media. While efforts have been made to help reduce the spread of misinformation on social media, many platforms are still largely unregulated. The influence of social media use on vaccination promotion is not fully understood. This systematic review aims to identify facilitators and barriers associated with vaccine promotion through social media use. *Materials and Methods:* Reviewers analyzed 25 articles and identified common themes. Facilitators of vaccine promotion included an increase in the efforts of social media companies to reduce misinformation, the use of social media to spread information on public health and vaccine promotion, and the positive influence towards vaccinations of family and friends. *Results and Conclusions:* Identified barriers to vaccine promotion included the spread of misinformation, decreased vaccine acceptance among users of social media for COVID-19 related information due to polarization, and a lack of regulation on social media platforms. The results of this review provide insight for improving public health campaign promotion on social media and can help inform policy on social media regulation and misinformation prevention.

## 1. Introduction

### 1.1. Rationale

The consequential Coronavirus (COVID-19) pandemic has heavily impacted the United States’ public health efforts. The high prevalence of COVID-19 cases and associated mortality rates resulted in the rapid development, production, and distribution of vaccines. While the healthcare industry has made significant gains in terms of technological advances and vaccine development, there are still plenty of challenges that need to be addressed within the current social context. The topic of vaccination remains controversial in the United States. Conspiracy theories and beliefs have increased vaccine hesitancy and led to stagnation in vaccination rates [1]. Conspiracy beliefs present a challenge in spreading accurate information regarding the COVID-19 pandemic and vaccines. These beliefs are also associated with a reduction in reported compliance with mitigation recommendations such as mask-wearing [2]. An individual’s choice related to a delay in vaccination (hesitancy) or otherwise completely resist vaccination is of great interest as the United States and rest of the world strive for herd immunity against COVID-19.

Social media has increasingly played a significant role in acting as a bank of information; however, it comes with its own benefits and downsides as a result of variation in regulation and the widespread sharing of individual perspectives [3]. Social media platforms are catered towards individuals and controlled by the same individuals. Even as these platforms work to improve features such as fact-checking and reliable resource compilations, it is difficult to identify and reduce the spread of misinformation. With the current COVID-19 pandemic, social media has played a large role in both vaccination promotion and relegation.

### 1.2. Objectives

It is important to discuss the various implications that social media has on the public’s views regarding the COVID-19 vaccine acceptance. Individuals are affected by social media in different ways, and the impact social media has, while not fully understood, is important to address. Social media can act as a facilitator or barrier when it comes to deciding whether a vaccine is the “right choice” [4,5]. The purpose of this review is to identify common facilitators and barriers in the literature which influence the promotion of vaccination against COVID-19. To encourage vaccination in the population and achieve herd immunity, policy makers, public health officials, and community leaders must identify ways to reduce barriers to vaccine promotion on social media.

This systematic review provides an objective assessment of publications in quality peer-reviewed journals as related to social media facilitators and barriers of COVID-19 vaccination status in the U.S. Social media platforms continue to be both scrutinized and celebrated based upon perceptions of dissemination of misinformation and freedom of speech protections in the U.S., while at the same time policymakers continue to entertain regulations to prevent the spread of inaccurate information related to the pandemic. In the end, a codification and classification of both facilitators and barriers related to vaccine promotion will provide policymakers with important information to better understand the public’s use of social media during the pandemic further support vaccination and overall herd immunity.

## 2. Methods

**Figure 1:**
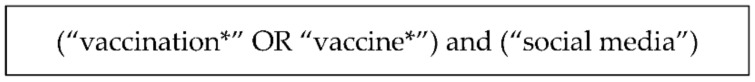
Research database search string and Boolean search operators that yielded the highest frequency of results in the search.

This systematic review was guided by the Preferred Reporting Items for Systematic Reviews and Meta-Analysis (PRISMA). Research articles included in this review were required to focus on the use of any variety of social media platforms and their discussion and dissemination of information regarding any COVID-19 vaccines. Researchers focused on the promotion of vaccines on any/all social media platforms using the research database search string shown in Figure 1.

The “vaccine” and “vaccination” terms in the search string were truncated (*) in the database search to allow for various uses of the terms in the review articles identified (plural uses of the terms, etc.). The identified search string, while somewhat broad, was quite effective in yielding the highest search results by remaining limited and all-encompassing of various research articles focusing on both vaccine and social media variables in the aggressive search criteria timeline. Further, this review criteria did yield articles focusing on other vaccinations (example: MMR, HPV, chicken pox), which were then later excluded by the research team. This approach helped ensure a more inclusive search for COVID-19 vaccinations as associated with social media platforms in the literature.

### 2.1. Eligibility Criteria

The search process specifically excluded any COVID-19 treatment medications or other therapies being investigated to meet the research team’s initiative surrounding the COVID-19 vaccine only. Any/all COVID vaccines in the United States (Pfizer, Moderna, and/or Johnson & Johnson) were included in the study. Articles had to be published in quality peer-reviewed journals and available on the institution’s the Ebson B. Stephens Company (EBSCO host) and PubMed (which queries MEDLINE). Ultimately, a total of three research databases were utilized in the search that a) increased the number of total search results for the review, while b) eliminating overall duplicate article findings: Academic Search Complete, MEDLINE, and Complementary Index. While some articles in the review identified and cited facts regarding the vaccinations and related distribution processes, validity of individual articles was not assessed by the research team in an effort to be as inclusive as possible regarding the review of uses and related observations via social media and the vaccines.

Articles included in the review were assessed for strength of evidence by utilizing the Johns Hopkins evidence-based practice rating scale (JHNEBP), a tool used to assist in clinical decision making which includes an evidence appraisal step to determine strength of evidence (articles in the review). Articles included in the review had a publication date within the 1 January 2020, to 2022 research database publication date range. The search was conducted by the research team from 10 July 2021, to 15 July 2021. Full text was not included as an initial search criterion (database search and related article identification) in order to yield as many search results as possible. Identification of full-text versions of each identified research article was later accomplished by the research team for all articles identified for the review (100% of the articles identified in full-text format by the research team).

This study’s information came from secondary data sources (library research database). All literature included in this research are publicly available and any individual research subjects (if present) are unidentifiable. As a result, this systematic review qualifies under “exempt” status in 45 Code of Federal Regulations (CFR) 46. An institutional review board review was not required, and no consent was necessary.

### 2.2. Exclusion Process

**Figure 2:**
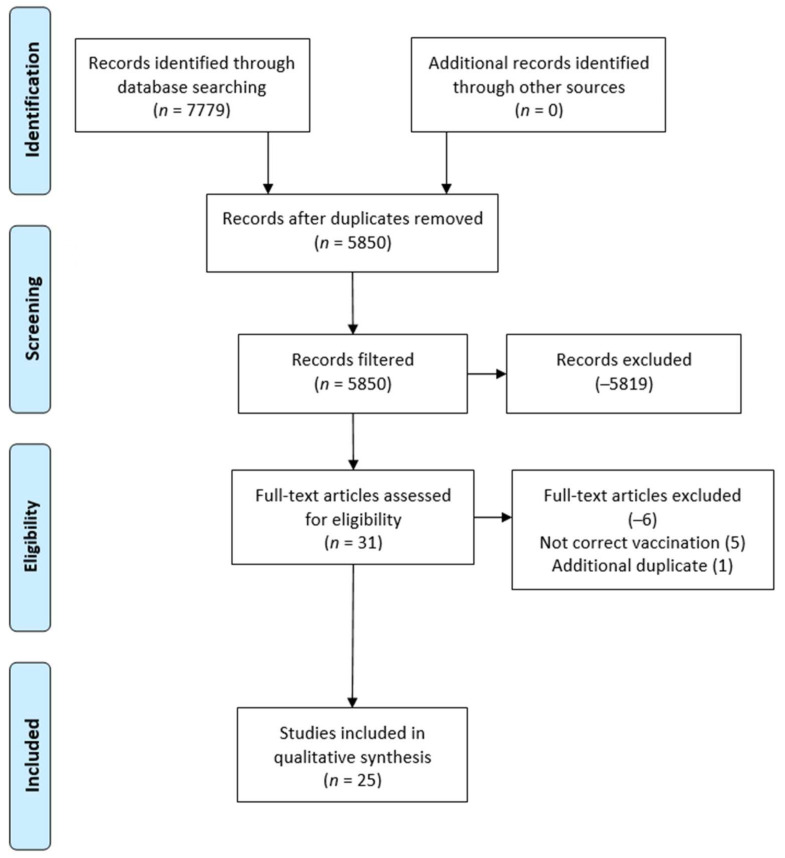
Preferred reporting items for systematic reviews and meta-analysis (PRISMA) figure that demonstrates the study selection process.

Figure 2 illustrates the article exclusion process. The initial research database search yielded 7779 results and the review team concluded the search and exclusion process with a final literature sample of 25 articles. While a small amount of literature was identified for review, the narrow search parameters helped to isolate only COVID-19 related vaccination articles associated with social media use. Further exclusion included the use of the research database’s “U.S. only” study option (checkbox), therefore helping to further identify studies that focused only on the area of interest for this study, as related to the U.S. vaccines and the country’s use of social media.

The research team also identified a large range of COVID-19 vaccination articles in the initial search queries (which did include preliminary Google search engine use) but was lowered when the social media construct was added to the search string.

A rigorous review of the 25 articles was conducted by the authors by reading the full manuscripts of each article. This was accomplished by an internal numbering of the articles and all researchers reviewing all 25 manuscripts collectively, with each article being reviewed by two or more researchers (Table 1). Next, reviewers split into two separate groups to review the assigned articles to identify the underlying themes related the facilitators and barriers of vaccine promotion during the COVID-19 global pandemic. Researcher collaboration meetings were conducted via webinar on multiple occasions and there were no disagreements in article coding and construct identification among the team members throughout the review.

## 3. Results

Reviews conducted by the research team consisted of a systematic approach to identifying underlying characteristics associated with the use of social media serving as a facilitator and/or a barrier to the COVID-19 vaccine in the United States during the global pandemic. In addition to the JHNEBP study design analysis, both facilitators and barriers with regard to the are summarized in Table 2. Articles are listed in alphabetical order by the first author’s last name.

### Risk of Bias

JHNEBP quality indicators were assigned to each article by the research team during the review process. While a majority of the review’s findings fall under level III (non-experimental, qualitative in nature) the inclusion of these articles was decided upon as it added to the quality of the review and identified, underlying constructs. There remaining five articles identified by the review are codified and shown in Table 3.

While it is preferred that research articles with strength of evidence ratings of level I and/or II are utilized in any systematic review, the researchers immediately identified a lack of published research in this segment of the U.S. healthcare industry to-date. As a result, all JHNEPB strength of evidence classifications were included in this study—to include level V (1 article). This decision was made to help ensure an adequate number of articles to review, while also ensuring the inclusion of both qualitative studies and some (3 articles) expert opinions regarding COVID vaccinations and social media.

**Figure 3:**
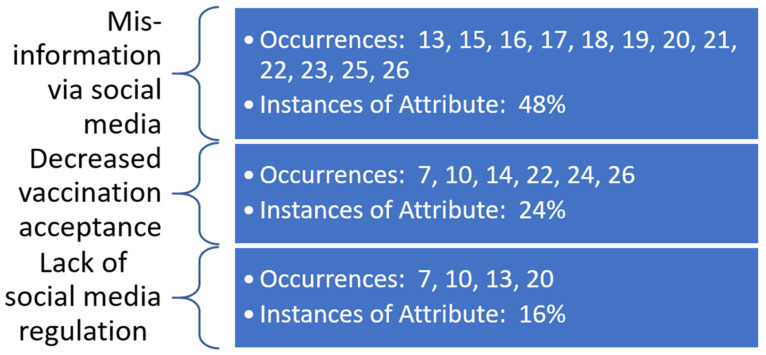
Occurrences of underlying themes identified as barriers to COVID-19 vaccine promotion in social media as observed in the literature.

**Figure 4:**
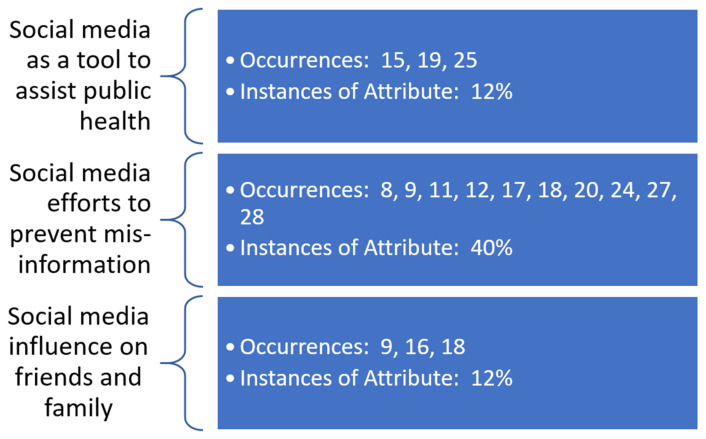
Occurrences of underlying themes identified as facilitators to COVID-19 vaccine promotion in social media as observed in the literature.

Underlying constructs related to both facilitators and barriers to the promotion of the COVID-19 vaccine via social media outlets were identified by the research team. The constructs are identified with meta-data shown in Figure 3 and Figure 4. These constructs are demonstrated to overlap, with multiple articles from the review supporting more than one underlying construct. This lack of construct exclusivity demonstrates the ability of social media to affect COVID-19 vaccine promotion in multiple ways.

The most evident underlying construct in this review was the use of social media to serve as an outlet for misinformation related to the COVID-19 vaccine—both as a barrier to vaccine promotion and as a facilitator to prevent misinformation related to the vaccine. Barriers related to decreased vaccine acceptance and lack of social media regulation were identified by the review team as further serving as barriers to vaccine promotion via social media outlets. Use of social media as a tool to assist various public health organizations and authorities and its influence on family and friends’ choices to undergo COVID-19 vaccination were also identified as additional facilitators to promotion via social media.

## 4. Discussion

### 4.1. Barrier: Misinformation Is Spread on Social Media and Often Contradicts Factual Health Information

One of the largest barriers to vaccine promotion through social media during the COVID-19 era has been misinformation spread on social media. Social media gives an outlet to everyone to express thoughts and feelings. This can be a great way to get information around to the public but the misinformation that can be spread is equally detrimental. Over the last several years many people have become victims to misinformation and have opted out of vaccinations due to reasons they found on the internet [26]. Unfortunately, vaccination rates are often below target. Much of this can be traced back to disproven studies that showed side effects such as getting autism, or a current disease being fought from a vaccine [30]. As the success of vaccines has been increasing for many years the public now has the luxury of choice. Due to past vaccines, once common diseases such as polio and measles are no longer a major threat. This can give people perceived safety and allow for misinformation to skew social norms of vaccination [22]. With the introduction of the COVID-19 vaccine misinformation has increased throughout social media. Rumors even discuss the possibility of microchips in the COVID-19 vaccines and suggest that the government is using the vaccine to track people [27]. Additionally, one study showed that high quality, verified information from sources like the Centers for Disease Control or the World Health Organization is often shared less than information from low value sources [28].

As vaccinations continue to roll out it will become critical to keep misinformation and conspiracy theories at bay. Many sites such as twitter and Facebook do not directly monitor these falsehoods which can be detrimental to the acceptance of the COVID-19 vaccine and putting a stop to the virus [30]. As vaccine hesitancy grows, social media can either be a tool to encourage greater protection via the COVID-19 vaccine or continue to fill knowledge gaps with misinformation preventing vaccination [17].

### 4.2. Barrier: Social Media Use for COVID-19 Information Results in Decreased Vaccine Acceptance Due to Polarization

Social media was widely cited as one of the barriers facing vaccine promotion efforts today. The percent of U.S. adults who use some form of social media has roughly doubled from 36% in 2009 to 72% in 2019, making these platforms one of the easiest ways to spread information [25]. Leading up to the COVID outbreak, other vaccination efforts found social media consumption to be a good predictor of vaccination intention [24]. During the COVID-19 pandemic specifically, studies show that social media is contributing to the spread of misinformation about the vaccine, and that individuals who were hesitant about the vaccine were more likely to only use social media as their source of news [28,30]. One study also found that people who solely obtained their news from social media, were more likely to believe in conspiracy theories surrounding COVID-19 and the vaccine itself when compared to those who consumed their news from traditional media outlets [30]. As the impact of social media continues to grow, it will become increasingly important for vaccination campaigns to overcome the spread of misinformation and polarization.

### 4.3. Barrier: Lack of Regulation of Social Media

Generally, social media tends to be self-regulating. However, due to a lack of regulation, a lot of vaccine skepticism can spread via social media. This can particularly affect the COVID-19 vaccine acceptance rate among individuals. In the past, Facebook has been associated with several violent outbreaks around the world because of heated discussions that occurred about vaccines via its platform [10]. Another example includes the public spreading information via social media websites about relying on herd immunity to be safe; these online discussions convince individuals that they are safe without choosing to be vaccinated [26].

Historically, the public has had extreme opinions on matters such as vaccination; therefore, a lack of social media regulation can result in harmful consequences to the public in relation to COVID-19 vaccine acceptance. Moreover, bots can also spread incorrect vaccine information via social media apps such as Twitter [13]. With a lack of regulation, bots spread misinformation, which in turn hinders the public’s judgement on the matter. While social media acts as a platform for individuals to openly share their thoughts, without proper regulation, it can be harmful to the public [10,26].

### 4.4. Facilitator: Social Media Used as a Tool to Share Information on Vaccine Promotion and Public Health

As social media continues to rise in popularity, it has the potential to be an effective source of public health information that is accessible and up to date [21]. Analyses of targeted online sentiments have allowed researchers to determine the efficacy of vaccine promotion efforts and alter initiatives to effectively disseminate crucial information [19,21]. In one study, researchers in Canada produced an analysis of Twitter sentiment which could be used by public health officials to distinguish messages that resonate more with users [19]. Moreover, social media platforms including Twitter, Facebook, and Instagram continue to be easily digestible sources of public health information for many laypeople [12]. For these platforms, the presence of vaccine opposition is apparent. Going forward, continual adaptations of rhetoric will be important to successfully deliver evidence-based vaccine guidance while combating the spread of vaccine misinformation [12,19,21].

### 4.5. Facilitator: Social Media Companies Increasing Efforts to Reduce Misinformation

Advancements in technology have improved the dissemination capabilities of social media platforms allowing a single post to reach millions of people. This has proved problematic, especially surrounding vaccines. Anyone can post on these platforms and are often not properly educated about the topic, causing misinformation about vaccines to be circulated causing distrust in their benefits [15,30]. “Vaccines should not become victims of their own success” [22]. Social media platforms are increasing their efforts to reduce the amount of misinformation by “limiting the untrue information and directing people to evidence-based websites” (i.e., the CDC) [22]. Findings from one of the studies selected indicate that two-sided refutational messages can be a promising strategy to combat vaccine misinformation [15]. Studies have suggested that providing more elaborated refutational messages “can reduce misperceptions because they help people understand the flaws of misinformation” [15]. 

### 4.6. Facilitator: Social Media Facilitation of Friends and Family Influence on Vaccinations

Social media is widely seen as an online extension to social circles. Research has found that online sharing of vaccine-related information among friends and family increases access to information and may positively impact individual willingness to receive a vaccine [9]. Individuals actively seeking out information regarding vaccinations may be more likely to trust the endorsement or personal story of a friend or family member. In addition, those with social networks endorsing intentions to receive a vaccination, were more likely to endorse vaccination intentions themselves [23]. The reverse was found to be true among those with peers who did not express intentions towards vaccinating. This effect is especially found among younger generations who tend to actively seek out healthcare information from online social circles [21].

## 5. Study Limitations

As with any review, this study possesses limitations. This was a convenience sample taken from articles focused on the U.S. only to provide an early identification of facilitators/barriers attributed to promotion of the COVID-19 vaccine through the use of social media platforms. Assessment of congruence across other non-U.S. healthcare industries was not conducted in this research and limits the external validity of the results to an extent. This search parameter was applied to address vaccine promotion facilitators and barriers specific to the United States due to the uniqueness of this country’s healthcare system and potential use and interpretation of online/social media venues.

Additional limitations involve the ongoing use of social media for COVID-19 vaccine promotion initiatives, both as facilitators and barriers communicated to the U.S. public. Due to the vast array of research methods among studies included in this review, a further assessment and/or classification of each article’s study design may demonstrate valuable information surrounding the mining of information from social media platforms. An identified area for future research involves analysis of misinformation and how it may be disseminated across social medial platforms, potentially using a time-series analysis that delineates among policy implementation dates, COVID-19 variants, and even new vaccines and therapeutics. Finally, ongoing investigation into the identified constructs may assist with understanding the rate of vaccinations across the county and how such details may not only assist the U.S. with ending the COVID-19 pandemic, but also assist other countries as they work towards this public health initiative as well.

## 6. Conclusions

The uncertainty regarding the COVID-19 pandemic continues to facilitate the spread of information across various platforms. Several facilitators and barriers were identified regarding COVID-19 vaccination acceptance and the use of social media. While social media can be used as a public health tool to spread accurate, scientifically supported information on COVID-19 vaccines, it can also be used as a platform to share misinformation. The literature shows that the spread of misinformation is a considerable barrier to vaccine promotion and those using social media for COVID-19 information are less likely to accept the vaccine. Future research should focus on facilitators of COVID-19 vaccine acceptance in the international setting to increase external validity by identifying strategies to reduce vaccine hesitancy in the United States. Policymakers and public health workers should encourage increased regulation of social media to help prevent the spread of misinformation about COVID-19 vaccination.

## Figures and Tables

**Table 1:** Reviewer assignment of the initial database search findings (full article review).

Article Assignment	Reviewer 1	Reviewer 2	Reviewer 3	Reviewer 4	Reviewer 5	Reviewer 6	Reviewer 7
1–10	X	X	X	X	X	X	X
11–20	X	X	X	X	X	X	
21–25				X	X	X	X

**Table 2:** Summary of Findings (*n* = 25).

Author(s)	Participant(s)	* JHNEBP Study Design	Facilitators of COVID-19 Vaccine Promotion on Social Media	Barriers to COVID-19 Vaccine Promotion on Social Media
Alshaabi et al. [6]	Twitter users	3	COVID has been heavily discussed among users of all languages Multiple peaks of attention/space/awareness identified to support vaccination	Language barriers (to include health literacy challenges) Accessibility to accurate data cited as a challenge (ex. government websites not utilized/cited) Accessibility to vaccine itself in developing areas of the world
Bonnevie et al. [7]	1000 publicly available Twitter posts related to vaccine opposition and posts were categorized into themes	3	Because of the debate, the topic is gaining more attention as the virus spreads Vaccine supporters might be more prone to getting vaccinated sooner	Vaccine opposition increased by 80% based on thread comments 12 different conversation themes identified Mistrust in health authorities observed in many threads WHO classifies vaccine opposition as one of the largest threats to public health
Bonnevie et al. [8]	117 influencers generated 69,495 engagements	3	Vaccination campaigns using a ground-up rather than top-down approach can feasibly reach at-risk groups with lower vaccination rates Shows the potentials of using an influencer-based model to communicate information about flu vaccination on a large scale	Used micro-influencers only so less follows of these type of social media threads Not reaching as many people as major influencer (celebrity) threads
Brandt et al. [9]	Two undergraduate classes (*n* = 58) at a public university in the southeastern part of US	2	College students are very active on social media Participants (97%) interacted on Facebook by “liking” a post or comment or posting a comment Participants demonstrated robust engagement and high treatment satisfaction Results suggests that social media is an effective platform to reach college students with health promotion interventions and increase HPV vaccination awareness in this important catch-up population.	Facebook is not as widely used among younger crowd as other apps such as Instagram, Twitter, and Snapchat Harder to reach a large young crowd on Facebook
Broniatowski et al. [10]	288,175 posts from 204 Facebook pages	3	Activity in pages promoting vaccine choice as a civil liberty increased in January 2015, April 2016, and January 2019 The 2019 increase was strongest in pages mentioning U.S. states	Discussion about vaccine safety decreased while discussion about civil liberties increased The Disneyland measles outbreak drew vaccine opposition into the political mainstream, followed by promotional campaigns conducted in pages framing vaccine refusal as a civil right
Budenz et al. [11]	Twitter messaging addressing gay, bisexual (GB) and other men who have sex with men (MSM)	3	Prevention/protection messaging was prevalent only in MSM tweets (49%) HPV vaccine sentiment was positive in GB + MSM messaging, there were deficits in volume of messaging Opportunity to shape vaccine uptake through PH agenda setting using social media messaging that increases knowledge and minimizes HPV vaccine stigma	There were deficits in volume of messaging, lack of focus on vaccination, and a proportion of negative tweets
Chan, et al. [12]	Twitter users; geolocated tweets from U.S. counties (N = 115,330)	3	Social media have demonstrated strong associations with vaccine patterns Programs to promote vaccination should encourage real-life conversations about vaccines	When the associations are negative, discussions with family and friends appear to eliminate them
Dunn et al. [13]	Sampled 53,188 U.S. Twitter users and examined who they follow and retweet across 21 million vaccine-related tweets	3	36.7% users retweeted vaccine-related content Only a small proportion of vaccine-critical info that reaches active U.S. Twitter users comes from bots	2.1% users retweeted vaccine content from bots 4.5% of users retweeted vaccine-critical to herd immunity comments
Dyer [14]	Eligible COVID-19 vaccine recipients	5	A proper compensation program may be a cheap and straight-forward solution to neutralizing vaccine hesitancy and bring the pandemic to an early end	Social media posts play into existing concerns that many people might not accept the vaccine, as surveys indicate
Featherstone & Zhang [15]	Sampled 609 U.S. adult participants with 5 message conditions (2 misinformation messages, 2 corresponding two-sided refutational messages, and 1 control group)	2	Two-sided refutational messages can be a promising strategy to combat vaccine misinformation	Conspiracy and uncertainty framed misinformation messages decreased pro-vaccination attitude Effects were further mediated by emotions of anger Parental status and conspiracy beliefs did not moderate effects of the messages on vaccination attitude
Hussain et al. [16]	Over 300,000 social media posts related to COVID-19 vaccines	3	Used AI to analyze the public sentiments toward the COVID-19 vaccine Social media is where the data comes from; researchers can tell the response of the public based on these apps By knowing public sentiments, policy makers are more informed when making decisions	Significant amount of this article is not about promoting the vaccine; it’s about finding out the how the public feels about the vaccine Public confidence can be shaken by misinformation about vaccine safety
Hwang [17]	48,600 people/random sample	3	Vaccinations were more likely if friends or families vaccinate their children	Misinformation online produces skepticism The more trust people put in social media, the more it will skew their view of vaccines
Jang et al. [18]	Canada vs. the U.S surveys on vaccination perceptions	3	This information can assist public health authorities in the monitoring and surveillance of health information, concerns, and behaviors, and can help tailor the public health strategy to the population Public health interventions on social media can break up the misinformation	Misinformation on social medial regarding vaccines beyond public health organizations’ controls
Khubchandani et al. [19]	Individuals were asked about their likelihood of getting the COVID-19 vaccine (asked before the vaccines came out). Questionnaire shared on social media and in personal groups.	3	Questionnaires and information can be shared on social media easily and disseminate quickly	Conspiracy theories and misinformation able to be identified/coded
Latkin et al. [20]	Study participants were recruited through Amazon’s Mechanical Turk (Mturk) service. Asked about their likelihood of getting the COVID-19 vaccine.	3	In-person communication is risky so more social media communication is encouraged Encourage a collective social identity around COVID-19 prevention, which may influence behavior change and increase vaccine acceptance among friends and family	Contradictory messages from the CDC and White House
Lin et al. [21]	515 valid cases from a sample of undergraduate students from a class at a large Northeastern University	3	The more college students rely on social media for H1N1 information, the more likely they are to be vaccinated When online news is not the dominant information source, dependence on social media sources is a significant variable in explaining potential vaccine uptake	Better understanding of the H1N1 threat does not necessarily motivate a stronger risk-prevention commitment
Nowak et al. [22]	716 members of the RAND American life panel with children under the age of 21 years were invited to take the survey. 615 responses.	3	A lot of information is out there on social media. Participants asked about knowledge and beliefs on 35 categories, political conspiracies, health conspiracies, putative vaccine side effects, gestalt vaccine endorsements, vaccine biology/epidemiology Twitter data was pulled related to the 35 categories	Prevalent misinformation on vaccines The people who post about issues on twitter are the ones who care about specific issues, causing polarization on the subject of vaccines
Oehler [23]	n/a	4	To counter social media misinformation, we need to develop or enlist “social media influencers” for medicine in the same ways that corporations and other groups promote their celebrities, products, and services so successfully Communicating on healthcare topics via a practice Facebook page, starting a twitter feed, posting, or sharing accurate health-related information to a professional Instagram account, and volunteering medical interviews to local broadcast and print media can all improve our footprint as medically trained “influencers” and can bolster respect for our practices as well	Spread of misinformation amongst specific groups on social media platforms. Similarly, accurate public health information gets pushed behind unwarranted rumors Shared online testimonials about adverse reactions to vaccines. Sharing of misinformation undermining physician recommendations or health information Lack of peer-review standards on social media platforms Ability for anti-vaccination groups to spread misinformation to a broad audience Intentional use of medical product marketing to mislead consumers to believe misinformation Weaponization of anti-vaccination messages by “bots” Social media platforms unwilling to take action/responsibility for misinformation. Almost 90% of older adults (ages 50 years or older) have used social media to find and share health information Major medical organizations have placed very low priority in developing their social media presence
Piltch-Loeb et al. [24]	Random mobile application users *n* = 2650 users out of over 900 million	3	A majority of participants reported hearing positive information about the COVID-19 vaccine, primarily from local TV	Public Health information shared by credible, evidence-based sources compete with unverified sources on the largest internet platforms There was a statistically significant difference in vaccine acceptance among those who had exclusively gotten information only from traditional media (46.9%), only from social media (29.3%), or both types of channels (37.1%) Those who are less likely to get the vaccine are using social media as their sole source of information, or as at least one of their sources of information Vulnerability of social media channels to exploitation by bad actors
Raghupathi et al. [25]	9581 vaccine-related tweets	3	Talks about measles vaccine by tracking sentiment (not necessarily topical) The positive sentiments related to the existence of a vaccine for measles, the vaccine being effective and the vaccine actually saving lives	Higher percentage of negative tweets about vaccines compared to positive tweets A strong misinformation study In an empirical study of Facebook users, it was demonstrated that positive information gets disseminated fast but does not sustain as long as negative information
Romer & Jamieson [26]	U.S. residents (*n* = 840)	3	A recent survey of content on Twitter concluded that despite the large amount of misinformation on social media There is also a great amount of science-based information that circulates on those sites	Use of social media in March was also predictive of vaccination in July, with an overall negative indirect relation of −0.041 (2020)
Rosenbaum [27]	Opinion/discussion article. Discusses U.S. adults, physicians, and researchers	4	In response to these dangerous disinformation campaigns, social media companies have intensified efforts to label falsehoods and eliminate them	While people firmly opposed to all vaccines may be relatively few in number, they wield outsized influence, particularly on social media, over the undecideds A recent study of expressions of vaccine-related sentiments by 100 million Facebook users found that antivaccine clusters of people, though less numerous than pro-vaccine clusters, have a more central presence in large networks and interact with more undecided clusters
Ruiz & Bell [28]	804 adults compensated English-speaking adults living in the U.S. 53.6% female, ages 18–65+	3	n/a	Respondents relying on social media for information about COVID-19 anticipated a lower likelihood of COVID-19 vaccine acceptance People are increasingly turning to social media for information expanding the potential for disseminating harmful health-related information
Speaker & Moffatt [29]	Article described the scope of a National Library of Medicine Global Health Events web archive	4	n/a	Social media provides a wealth of misinformation and conspiracy theories
Zhang et al. [30]	2,598,033 tweets from 3 Twitter datasets	3	Social media platforms such as Facebook can be important tools of information regarding effectiveness of the HPV vaccine Same as above except this article analyzes tweets	Twitter algorithm investigation and discussion provides detailed insight into public responses to posts/tweets regarding vaccinations

* Johns Hopkins Nursing Evidence-Based Practice (JHNEBP) levels of strength of evidence: Level 1, experimental study/randomized control trial (RCT); Level 2, quasi-experimental study; Level 3, non-experimental, qualitative, or meta-synthesis study; Level 4, opinion of nationally recognized experts based on research evidence/consensus panels; Level 5, opinions of industry experts not based on research evidence.

**Table 3:** Summary of Quality Assessments.

Strength of Evidence	Frequency
II (Quasi-experimental)	2 (8%)
III (Non-experimental, qualitative)	19 (76%)
IV (Opinion of nationally recognized experts based on research evidence/consensus panels)	3 (12%)
V (Opinions of industry experts not based on research evidence)	1 (4%)

## Data Availability

Not applicable.
